# Treatment of the Developmental Dysplasia of the Hip with an Abduction Brace in Children up to 6 Months Old

**DOI:** 10.1155/2015/103580

**Published:** 2015-02-26

**Authors:** Raphaël Wahlen, Pierre-Yves Zambelli

**Affiliations:** Orthopedics Department, University Hospital of Lausanne, 1011 Lausanne, Switzerland

## Abstract

*Introduction*. Use of Pavlik harness for the treatment of DDH can be complicated for parents. Any misuse or failure in the adjustments may lead to significant complications. An abduction brace was introduced in our institution, as it was thought to be easier to use. *Aim*. We assess the results for the treatment of DDH using our abduction brace in children of 0–6 months old and compare these results with data on treatments using the Pavlik harness. *Method*. Retrospective analysis of patients with DDH from 0 to 6 months old at diagnosis, performed from 2004 to 2009. Outcomes were rates of reduction of the hip and avascular necrosis of the femoral head (AVN). Follow-up was at one year and up to 4 years old. *Results*. Hip reduction was successful in 28 of 33 patients (85%), with no AVN. *Conclusion*. Our results in terms of hip reduction rate and AVN rate are similar to those found in literature assessing Pavlik harness use, with a simpler and comfortable treatment procedure.

## 1. Introduction 

Developmental dysplasia of the hip (DDH) is defined by acetabular dysplasia, which can lead to hip dislocation. For children from 0 to 6 months old with a confirmed DDH, the position for treatment is bending and abducion of the hips, using a restraining device such as Pavlik's harness [1, 2]. This harness was developed to improve hip reduction rates and reduce the incidence of femoral head avascular necrosis (AVN). Unlike other methods, such as the von Rose braces, the Becker, and Mittelmeier abduction pillow or other rigid abduction braces, Pavlik's harness is a dynamic positioning device that avoids complete immobilization of the joint [3–7]. The absence of external constrains allows gradual and gentle reduction and stabilization of the hip. The success of Pavlik's method is now well established [8–12].

However, AVN has not disappeared and remains a serious complication. Furthermore, failure of hip reduction can still occur with the harness [13–15]. Other complications as femoral neuropathy, brachial plexus neuropathy, knee subluxation, or skin lesions have also been described [5, 7, 16]. According to Mubarak et al. [16] these mostly occur following inappropriate medical indication or misuse of the harness. A lack of treatment compliance by parents can be a challenging cause of failure. The use of Pavlik's harness requires regular medical check-ups, frequent technical adjustments, and continuous parental monitoring. It puts pressure on parents in terms of both costs and compliance [16, 17].

In 2003, Hedequist et al. [18] reported encouraging results with the use of an abduction brace when stabilization with the Pavlik harness failed. They also valued the ease of application for the parents and therapist. Following this publication, we decided to replace the use of the Pavlik harness with a custom made abduction brace for the treatment of DDH in children up to 6 months old. The brace would be easier to use than Pavlik's harness but would preserve the therapeutic advantages and characteristics, which contribute to its success. The objectives of this study were to assess the brace's efficacy and safety, in terms of hip reduction rate and AVN rate, and to compare our results with those of Pavlik's harness.

## 2. Materials and Methods

We performed a retrospective review of the medical records of all the children from 0 to 6 months old at diagnosis with clinically and imaging confirmed DDH. They were treated in 2 hospitals close to our institution from 2004 to 2009. Children with DDH secondary to neurological, myopathic, or connective tissue diseases were excluded; two other patients were excluded because of data loss. In one patient with Graf 4 dysplasia, we decided to immediately initiate a closed reduction with cast, and so he was also excluded from the study. All the patients had been treated by the same surgeon, and ultrasound (US) follow-up evaluations had been performed by trained radiologists. No other methods were used as first-line in this age group.

We looked at 40 dysplastic hips affecting 33 patients. Seven dysplastic hips were bilateral. We reported on 10 Graf type 2a hips, 12 type 2b hips, 8 type 2c hips, 7 type 3 hips, and 1 type 4 hip. One patient had a normal hip—Graf US type 1—despite a clearly pathological status. Another 6-month-old infant did not receive a US examination but a pelvis X-ray instead and thus was not classified using the Graf method. All 33 patients had a follow-up at 1 year old and 28 patients at 3-4 years old (85%).

Systematic clinical assessment of the hips was performed at birth. A negative clinical examination at birth, without risk factors, leads to the routine clinical follow-up at 4–6 weeks old. In case of a positive risk factor (positive family history, breech delivery, oligoamnios, and twins), US evaluation was performed at 4–6 weeks old. In cases of positive clinical findings for hip luxation at birth, a US evaluation of the hip joint was performed at 1-2 weeks old. At this time, fully dislocated hips were splinted. Immature hips were controlled with US follow-up 1-2 and 4–6 weeks later, as normalization within the first weeks of life is frequent [19, 20]. The brace treatment was only initiated in confirmed DDH after this time. Inconclusive examinations were reassessed every 2 weeks until a final diagnosis was made (Table 1).

Our decisions to treat DDH with an abduction brace were based on both clinical evaluation and results of the imaging studies (Table 2). The US diagnosis of DDH was based on the Graf hip classification, which uses *α* and *β* angles, as well as the acetabular coverage, which was estimated at higher or lower than 50%.

**Figure 1:**
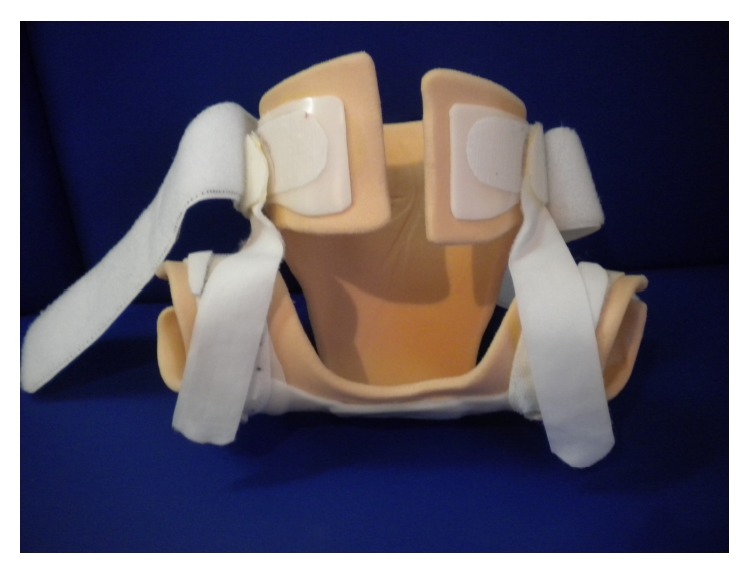
Brace, view from front.

**Figure 2:**
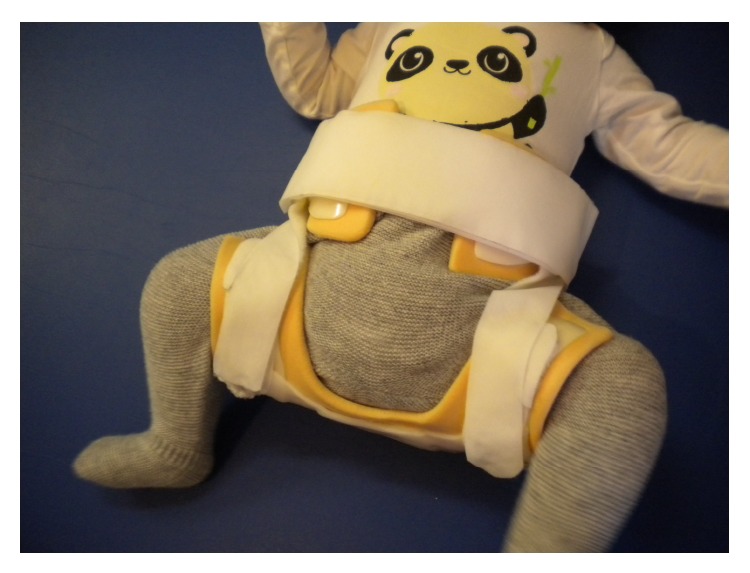
Brace on the child, view from front.

**Figure 3:**
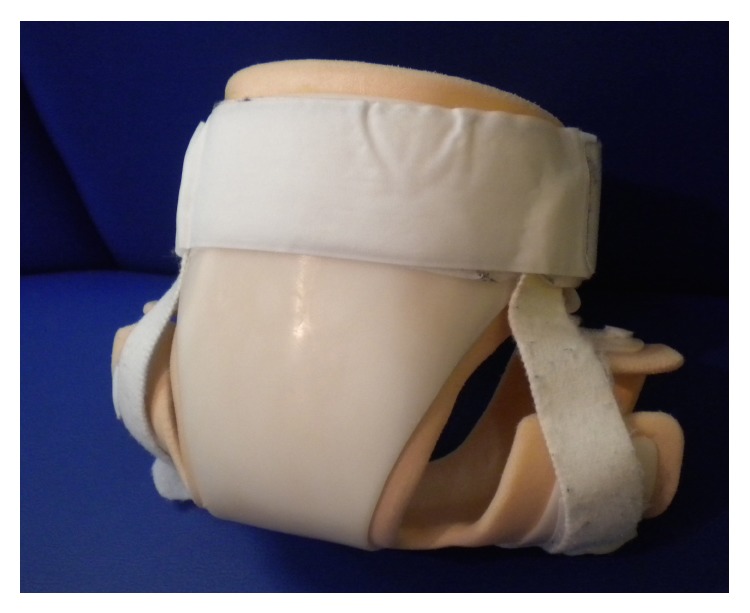
Brace, view from behind.

**Figure 4:**
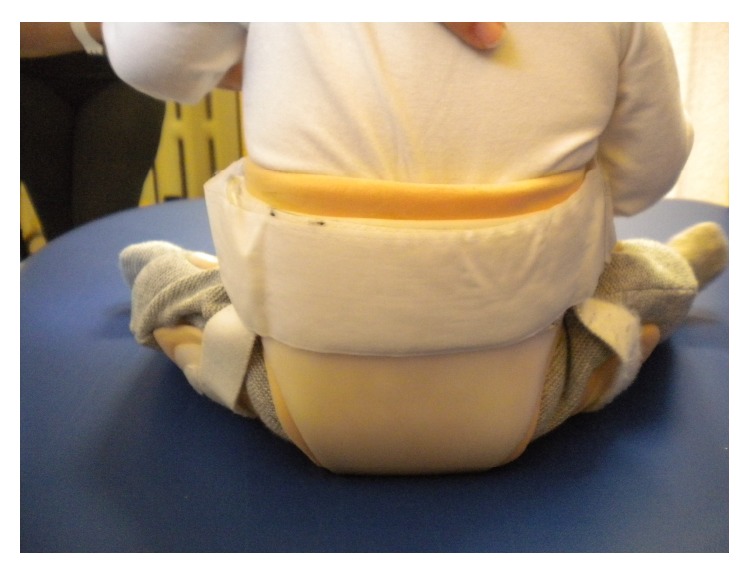
Brace on the child, view from behind.

The custom made brace used in Lausanne was conceived following Hedequist et al.'s publication. Initial prototypes were made based on Hedequist's design, but through a collaborative process between physicians, occupational therapists, and parents, we arrived at our current improved device (Figures 1, 2, 3, and 4). The brace design comes as close as possible to a dynamic positioning orthosis. It thus differs from earlier abduction splints, which completely immobilized the hips and, therefore, caused a high rate of AVN [7].

Plastazote foam, a quite flexible material allowing some degree of mobility, is molded on the infant's body from the level of the armpits to the low pubis and enables splint adjustment to the size of each individual newborn. Thighs are embraced, but the bending area is kept free for limb movements; knees are free of constraint and are able to move. The internal hull is reinforced by a second, external plastic layer, covering the back and thigh areas and is made of aquaplast, a semirigid foam. This can be tightened should the newborn be too active. In order to reinforce hip's bending and abduction, more rigid material can be added around the buttocks area. Flexion and abduction, between 90° and 100°, are adjustable through a rigid strap at the front of the body and through an elastic strap at the back. The back strap usually allows maximal abduction amplitude. This elastic strap and the flexibility of plastazote prevent a rigid hip immobilization which might alter the vascularization of the femoral head.

At early stages of treatment, the physician's recommendation was usually “let's try it and see,” without any undue hurry for the perfect position. This was followed by progressive moves towards a perfect fit, through heating and molding the brace. In this way, we ensured that hips were gradually reduced without excessive hardship. During this period, the splints were removed daily, at bath and feeding times only, which still corresponded to 23 h wearing time per day. Follow-up consisted of a monthly medical examination and US. From 4 months old, US was replaced by pelvic radiography, and we assessed hip evolution by calculating the acetabular Hilgenreiner angle.

Medical examinations required a brief removal of the brace on a regular basis. If a positive response and stabilization occurred, we indicated a part-time treatment and night-time brace wear only. When the hip's evolution was not satisfactory, we proceeded towards arthrography, closed reduction of the hip, and cast. After this, the splints were worn part-time, at night, for 2 months. Another X-ray was taken at 1-year old to document any residual dysplasia or signs of AVN. We carried out further tests, clinical and radiological, at 3-4-years old. The criteria for success were satisfactory hip reduction and an absence of evidence of AVN at 1- and 4-year old. Finally, we compared our results with those in the literature about Pavlik's harness.

## 3. Results

The abduction brace allowed reduction in 28 out of 33 patients (85%) (Tables 3 and 4). In 5 cases, this method was insufficient; thus a closed reduction and cast became the next therapeutic option. All Graf types IIa, IIb, and IIc unilateral dysplasias were successfully reduced by our abduction brace and in 4 out of 6 unilateral type III (66%). The patient with Graf type IV hip was not successfully reduced. Reduction was achieved in 4 out of 7 of bilateral dysplasia (57%).

At 1-year old, 9 patients (27%) showed persistent dysplasia above standard intervals for their age, with higher Hilgenreiner angle, a slightly eccentric position of the femoral head, or rupture of the cervicoobturator line. Out of the 28 patients with a follow-up at 3-4-year old, 5 patients among the 9 patients with persistent dysplasia at 1-year old had hip normalization and 4 (15%) still showed residual dysplasia. Regarding AVN, none has been detected and no other complication due to the brace occurred.

It should be noted that since 2007, 23 out of a total of 24 patients (96%) were successfully treated with the abduction splint only. The only failure occurred in bilateral types IIc and IIb dysplasia. Out of these 24 patients, 5 residual dysplasias remained at the age of 1 year (21%). Out of the 19 patients with follow-up at 3-4-year old, 3 of the 5 patients with residual dysplasias at 1-year old had hip normalization and 2 patients had residual dysplasia (11%).

Regarding unilateral dysplasias (Table 5), the mean age of brace introduction was 3.1 months (range, 3 weeks old to 6 months old). The wearing time was full-time (23 h/day) during a mean 3.6-month period (range, 2 months old to 5 months old), except for the patients undergoing a secondary reduction. The brace was worn for a total mean period (first full-time, then part-time at night) of 5.3 months (range, 3 months to 8 months). Regarding bilateral dysplasias (Table 6), the mean age of brace introduction was 1.5 months (range, 1 week old to 3 months old). This treatment had to be interrupted and replaced in 3 out of 7 patients, after an average period of 3 months (range, 1 month to 5 months). Patients whose hips were reduced using the brace wore them full-time (23 h/day) over an average period of 6.3 months (range, 5 months to 8 months). Overall (first full-time and then part-time), the brace was worn for an average period of 8 months (range, 6 months to 10 months).

## 4. Discussion

Pavlik's harness is currently the favored device used to treat DDH. In literature, reduction rates obtained with this device range from 80% to 100% [9, 20, 22, 23]. The severity of DDH can affect the treatment success ratio. Indeed, in severe luxation, Pavlik's harness is often ineffective. Reduction rates with the harness for type 4 dysplasias are as low as 0% for some authors [24, 25], but better results are also obtained with 50% [26] and 62% [2] of successful reduction rates. The second important outcome is the AVN rate after treatment. In literature, commonly reported AVN ratios are between 0% and 8% [10, 23, 24, 27].

Our Lausanne-developed abduction brace reached similar therapeutic results to those currently found in the literature on Pavlik's harness. In fact, our splint raised reduction rates to 85% of the cases from 2004 to 2009 and up to 96% of the reported cases from 2007 to 2009. The increase in successful outcomes over the last 3 years may be attributable to improvements in the achievement of the custom made brace over time, on one hand, and to more numerous low-range and midrange dysplastic hips encountered, on the other. The ratio of residual dysplasia at 1-year old was 23.5% and 15% at 3-4-year old. The other key outcome was a 0% ratio of AVN. We had no reports of other complications like femoral nerve issues or skin lesions.

The brace is effective for low-range and midrange Graf types IIa, b, and c, with a 100% success ratio for unilateral dysplasia. With type III dysplasia, however, this ratio dropped to 71.4% (5/7). We were unable to reduce the Graf type IV case. These results were similar to those obtained with Pavlik's harness in some of the literature [24–26] but were less convincing for severe dysplasia than in Peled et al. study [2]. Bilateral dysplasias produced also a lower reduction rate of 57.1% (4 out of 7); they were often severe and hard to reduce using only a brace. It should be noted that the failure of brace treatment in no way affected any secondary treatment by traction and closed reduction.

The brace was developed to make treatment of DDH easier to manage for physicians and for parents and children in day-to-day life. Being made of light, flexible material, its daily use is very easy for parents. Moreover, our brace allows the infant to sit. Reshaping the brace during the treatment is very easily done by slightly heating and molding the thermoplastic material into the desired position. So with a splint lifespan of about 3 months, only two different braces are usually required for the duration of the treatment. One key element is that the design avoids any maladjustment for the user, unlike Pavlik's harness, so that we can manipulate the brace without any risk of accidents and associated serious consequences. In our country, the Pavlik harness is charged 400 Euros and the splint 600 Euros. However, while the harness requires a medical follow-up almost weekly, or every 2 weeks, the splint allows a follow-up every 3 weeks, alternately by the occupational therapist and the orthopedic surgeon.

Given the limited number of patients, we have not been able to build up significant statistics and figures and, therefore, to draw firm conclusions. One limitation to the use of the brace is the presence of well-trained occupational therapists to make the brace, which cannot be used in every clinic set-up, or a pediatric orthopedic office. However, in the light of our results, and given the ease of management compared to Pavlik's harness, we are continuing to use our abduction brace for the treatment of DDH in infants between 0 and 6 months old.

## Figures and Tables

**(a):** 

Clinical findings and risk factors (RF)			Code

(A) Dislocated	(1) Nonreducible		A1
	(2) Reducible		A2

(B) Nondislocated	(1) Unstable	(1.1) Unstable after 1-2 weeks	B11
		(1.2) Stable after 1-2 weeks	B12
	(2) Stable	(2.1) Positive RF	B21
		(2.2) Negative RF	B22

**(b):** 

Code	Management

A1	US + traction + cast
A2	US + abduction brace
B11	US + abduction brace
B12	US at 4–6 weeks, routine physical examination
B21	US at 4–6 weeks, routine physical examination
B22	Routine physical examination

**Table 2:** US results and medical management: algorithm (adapted from Mahan et al. [21]).

Age	Alpha angle	Graf type	Medical management		
<3 months old	>60°	I	Clinical follow-up		
	50–60°	IIa	Repeating US at 4–6 weeks old	Improvement	US follow-up
				Unchanged or worse	Treatment
	<50°	III, IV	Treatment		

>3 months old	>60°	I	Clinical follow-UP		
	<60°	IIb, IIc, III, IV	Treatment		

**Table 3:** Values of unilateral dysplasia.

ID	Graf	Side	Residual dysplasia at 1-year old	Residual dysplasia at 4 years old	Failed reduction	AVN	Comments
1	2b	G	0	0	0	0	
2	2b	G	0	0	0	0	
3	3b	G	0	0	1	0	Failure + cast
4	3	D	1	1	1	0	Failure + cast
5	3	G	1	0	0	0	
6	1	G	0	0	0	0	Normal image at birth but pathological clinic
7	2a	G	0	0	0	0	
8	2b	G	0	0	0	0	
9	3	G	0	0^*^(2)	0	0	
10	2b	G	0	0	0	0	
11	2b	G	0	0	0	0	
12	2a	G	0	0	0	0	
13	3	D	0	0	0	0	
14		D	1	0	0	0	
15	2a	G	0	0	0	0	
16	2b	D	0	0	0	0	
17	3	G	0	0^*^(3)	0	0	
18	2b	G	0	0	0	0	
19	2a	G	0	—	0	0^*^(1)	
20	2c	G	0	0	0	0	
21	2b	D	1	1	0	0	
22	2c	D	1	0	0	0	
23	2b	G	0	—	0	0^*^(1)	
24	2b	G	0	—	0	0^*^(1)	
25	3	D	0	0	0	0	
26	2c	G	1	0	0	0	

^*^(): age at last control.

**Table 4:** Values of bilateral dysplasia.

ID	Graf		Residual dysplasia at 1		Residual dysplasia at 4		Failed reduction	AVN
	D1	D2	D1	D2	D1	D2		
1	2b	2b	1	0	1	0	0	0
2	2c	2a	1	0	0^*^(3)	0^*^(3)	1	0
3	4	2c	0	0	0	0	1	0
4	2c	2a	0	0	0	0	0	0
5	2a	2a	0	0	0	0	0	0
6	2c	2a	1	0	1	0	1	0
7	2c	2a	0	0	—	—	0	0^*^(1)

^*^(): age at last control.

**Table 5:** Brace duration, unilateral dysplasia.

ID	Brace, introduction age (months)	Brace 23 h/day (months)	Total brace duration (months)	Secondary treatment
1	6	3	3	
2	3	4	5.5	
3	1	1	—	Traction, cast
4	4	3	3	Traction, cast
5	1.25	5	7	
6	6	3	6	
7	2.5	4	6	
8	3	2	5	
9	1	5	7	
10	3	4.5	6.5	
11	4	5	6	
12	0.75	4.5	7	
13	2.5	3	4	
14	6	4	6	
15	2.5	3	4	
16	3.5	3	6	
17	5	4	5	
18	3	5	8	
19	2	3	5	
20	1.5	2.5	5.5	
21	4	2	3	
22	2	4.5	6	
23	6	4	6	
24	3.5	2.5	5.5	
25	1.5	4	6	
26	1	5	6	

**Table 6:** Brace duration, bilateral dysplasia.

ID	Brace, introduction age (mo)	Brace 23 h/day (mo)	Total brace duration (mo)	Secondary treatment
1	3	7	9	—
2	0.25	5		Traction, cast
3	1	1		Traction, cast
4	1.5	8	10	—
5	1.5	5	7	—
6	2	3		Traction, cast
7	1	5	6	—
